# Therapeutic Drug Monitoring of Conditioning Agents in Pediatric Allogeneic Stem Cell Transplantation; Where do We Stand?

**DOI:** 10.3389/fphar.2022.826004

**Published:** 2022-03-07

**Authors:** M. Y. Eileen C. van der Stoep, Lisa V. E. Oostenbrink, Robbert G. M. Bredius, Dirk Jan A. R. Moes, Henk-Jan Guchelaar, Juliette Zwaveling, Arjan C. Lankester

**Affiliations:** ^1^ Department of Clinical Pharmacy and Toxicology, Leiden University Medical Center, Leiden, Netherlands; ^2^ Willem-Alexander Children’s Hospital, Department of Pediatrics, Leiden University Medical Center, Leiden, Netherlands

**Keywords:** conditioning regimen, TDM, pediatrics, HSCT, pharmacokinetics

## Abstract

Allogeneic hematopoietic stem cell transplantation (HSCT) is an established curative treatment that has significantly improved clinical outcome of pediatric patients with malignant and non-malignant disorders. This is partly because of the use of safer and more effective combinations of chemo- and serotherapy prior to HSCT. Still, complications due to the toxicity of these conditioning regimens remains a major cause of transplant-related mortality (TRM). One of the most difficult challenges to further improve HSCT outcome is reducing toxicity while maintaining efficacy. The use of personalized dosing of the various components of the conditioning regimen by means of therapeutic drug monitoring (TDM) has been the topic of interest in the last decade. TDM could play an important role, especially in children who tend to show greater pharmacokinetic variability. However, TDM should only be performed when it has clear added value to improve clinical outcome or reduce toxicity. In this review, we provide an overview of the available evidence for the relationship between pharmacokinetic parameters and clinical outcome or toxicities of the most commonly used conditioning agents in pediatric HSCT.

## Introduction

Allogeneic hematopoietic stem cell transplantation (HSCT) is an established curative treatment for malignant and non-malignant disorders in both adult and pediatric patients. In HSCT, the hematopoiesis of the host (i.e., the patient) is eliminated by a conditioning regimen in order to allow donor (i.e., healthy individual) stem/progenitor cell engraftment in the bone marrow and thymic niches. Furthermore, prevention of immune-mediated rejection is an important goal of conditioning regimens that should facilitate a successful HSCT outcome (Copelan 2006). Depending on the underlying disease, the conditioning regimen usually consists of agents that have myeloablative (MA) properties to create “space” in the bone marrow of the patient and eradicate the primary disease (Shaw et al., 2019). Immunoablative/-suppressive agents are applied to prevent rejection (host-versus-graft) as well as graft-versus-host disease (GvHD). After the infusion of the donor stem cells containing graft, immunosuppressive agents are usually used as prophylaxis to ensure engraftment and prevent the development of GvHD (McCune and Bemer 2016).

The choice for the optimal conditioning regimen is dependent on different factors. The required intensity of the conditioning regimen, particularly the immunosuppressive component, is usually greater when an unrelated or mismatched family donor is used. Myeloablative regimens are associated with a high likelihood to result in full donor chimerism, a situation where the newly developed hematopoietic system is of donor origin only (Bader et al., 2005). For malignant diseases, MA regimens are often required to eradicate all malignant cells, whereas in patients with non-malignant diseases less intense protocols can also be sufficient, depending on the specific disease and required level of chimerism. These less intense, non-MA protocols are often referred to as reduced intensity (RIC) regimens, in which the use of reduced doses of myeloablative drugs (or radiotherapy) is more likely to result in mixed chimerism, a state where donor and recipient hematopoiesis coexist within the recipient (Bader et al., 2005; Shaw et al., 2019). In addition, patient specific factors, such as age, immune status, DNA repair disorders, tumor load, disease activity and comorbidities, play a role in requirement for and tolerability to the various conditioning agents and therefore the choice for the preferred regimen (Nagler and Shimoni 2019). Nowadays, more emphasis is placed on the immunosuppressive aspect of the regimen to prevent rejection and GvHD in the case of unrelated or mismatched donors (Baron et al., 2017). While an effective conditioning regimen is necessary prior to the infusion of the HSCs, it may also be accompanied with acute toxicity which can even be life-threatening. Complications related to toxicity of the conditioning regimen are still a major cause of transplant-related mortality (TRM). Besides the risk of acute toxicity, late toxicities, such as infertility, are also a major problem (Diesch-Furlanetto et al., 2021). One of the main challenges to improve HSCT outcome is reducing toxicity caused by the conditioning regimen while maintaining efficacy.

In the last decade, significant improvements have been made to optimize efficacy and safety of conditioning regimens. These include the use of less toxic agents, less toxic combinations and dose optimization. Personalized dosing of several components of the conditioning regimen by means of therapeutic drug monitoring (TDM) has contributed to more favorable HSCT outcome. Therapeutic Drug Monitoring is the clinical practice of individualization of dosage by measuring plasma or blood drug concentrations and maintaining it within a therapeutic range or window. TDM is considered useful when the following criteria are met (Saha 2018): 1) There should be a clear relationship between concentration and effect (either efficacy or toxicity or both), 2) drug concentrations cannot be predicted from a given dose, because of high interindividual variability in pharmacokinetic (PK) parameters, 3) the drug has a narrow therapeutic index, 4) the dose cannot be easily optimized by clinical observation and 5) a bioanalytical assay should be available. TDM in combination with the use of mathematical models (such as population PK models), and other patient and disease characteristics, such as genotype, organ function, and age, is now increasingly being used to personalize dosing right at the start of treatment; a dosing paradigm that is now often referred to as ‘model-informed precision dosing (MIPD)’ (Darwich et al., 2021).

Especially in children, TDM/MIPD can be of value. Because of the development and maturation of organ systems, in general children have greater pharmacokinetic variability than adults due to age-related differences in drug metabolism (Kearns et al., 2003). Also, the developing organ systems may lead to different susceptibility to toxicity. Moreover, pharmacokinetic studies in children are sparse which makes it challenging to establish evidence based TDM recommendations. In this review, the focus lies on providing an overview of the available evidence for the relationship between pharmacokinetic parameters and clinical outcome or toxicities of the most commonly used conditioning agents given prior to pediatric HSCT and discuss whether TDM could be a useful tool to improve outcome.

## Literature Search Methods

Literature searches in PubMed were conducted using the generic names of the conditioning agents and the terms “pharmacokinetics” and “pediatric” (e.g., treosulfan AND pharmacokinetics AND pediatric). The results were screened and studies were included if the majority of patients were ≤18 years of age and if PK parameters of the drug were studied in relationship to toxicities and/or outcome. For busulfan, only the studies that report either a hazard ratio (HR), odds ratio (OR) or relative risk (RR) were selected to limit results and keep the review concise. For a detailed overview of busulfan PK studies we refer to two recent reviews (Ben Hassine et al., 2021; Lawson et al., 2021). Studies were described in chronological order.

## Chemotherapy

### Busulfan

Busulfan (Bu) is a widely used and established chemotherapeutic agent in conditioning regimens prior to HSCT. It is a bifunctional alkylating agent that diffuses into cells, where it is hydrolyzed to produce highly reactive carbonium ions that alkylate and damage DNA (Pierre Fabre Médicament, 2017). Its metabolism is complex and not yet completely understood. It is primarily metabolized by the liver through conjugation with glutathione, mainly by glutathione-S-transferase A1 (GSTA1). The glutathione conjugate is then further oxidized before it is excreted into the urine. Intravenous (i.v.) Bu has widely replaced oral Bu when this formulation became available, which was expected to reduce pharmacokinetic variability (Ciurea and Andersson 2009). However, interpatient variability in clearance of i.v. Bu is still reported to be up to 30% (McCune and Holmberg 2009; Lee et al., 2012). Factors explaining this interpatient variability in children are age, body weight and GSTA1 genotype, among others (Lawson et al., 2021). In the past decades, many studies have shown that Bu exposure is related to clinical outcome. In Table 1, the studies that report either a hazard ratio (HR), odds ratio (OR) or relative risk (RR) are shown.

**TABLE 1:** Reported associations of pharmacokinetic parameters of busulfan and clinical outcomes.

First author, year	*n*	Age, median (range)	Diagnosis	Regimen	Dose interval	Major findings
Bartelink et al. (2009)	102	3.1 (0.2–21.0)	Malignant: 46 (45%)	BuCyMel: 43 (42%)	Once daily: 64 (63%)	Bu exposure of 72–80 mg*h/L was associated with the highest OS and EFS (*p* = 0.021 and *p* = 0.028). Increased AUC was associated with less graft failure and relapse (HR 0.047; *p* = 0.004), but more aGVHD (HR 1.56; *p* = 0.019).
			Non-malignant: 56 (55%)	Other: 59 (58%)	4 times daily: 38 (37%)	
Ansari et al. (2014)	75	6.2 (0.1–20.0)	Malignant (ALL/AML/MDS): 48 (64%)	BuCy: 67 (89%)	4 times daily	C_ss,day1_ > 600 ng/ml (daily AUC of 14.4 mg*h/L or cumulative AUC of 57.6 mg*h/L) was associated with lower EFS (HR 5.14; 95%CI 2.19–12.07; *p* < 0.001) and lower OS (HR 7.55; 95%CI 2.20–25.99; *p* = 0.001).
			Non-malignant: 27 (36%)	BuCyVP16: 6 (8%)		
				BuMel: 2 (3%)		
Bartelink et al. (2016)	674	4.5 (0.1–30.4)	Malignant: 274 (41%)	BuCy: 352 (52%)	Once daily: 267 (40%)	Optimum cAUC of 78–101 mg*h/L decreased the probability of graft failure or relapse (HR 0.57; 95%CI 0.39–0.84, *p* = 0.0041). High cAUC increased the risk of TRM (HR 2.99; 95%CI 1.82–4.92, *p* < 0.0001) and toxicities (HR 1.69; 95%CI 1.12–2.57; *p* = 0.013).
			Non-malignant: 400 (59%)	BuFlu: 252 (37%)	4 times daily: 324 (48%)	
				BuCyMel: 70 (10%)	Other: 83 (12%)	
Benadiba et al. (2018)	36	5.9 (0.6–19.3)	AML: 23 (63.9%)	BuCy: 33 (91.7%)	4 times daily	C_ss,day1_ > 600 ng/ml (daily AUC of 14.4 mg*h/L or cumulative AUC of 57.6 mg*h/L) was a significant risk factor for OS (HR 5.2; 95%CI 1.26–21.5, *p* = 0.02) and EFS (HR 3.83; 95%CI 1.33–11.05, *p* = 0.01).
			MDS: 13 (36.1%)	BuMel: 2 (6%)		
				BuCyVP16: 1 (2.3%)		
Philippe et al. (2019)	293	6.2 (0.2–21.0)	Malignant: 170 (58%)	Not clearly specified	4 times daily: 282 (96%)	The incidence of VOD was 25.6%. Patients with C_max_ of ≥1.88 ng/ml were 6 times more likely to develop VOD (63.3 vs. 21.3%, RR 6.0 *p* < 0.001).
			Non-malignant: 123 (42%)		Once daily: 10 (3.4%0	
					Twice daily: 1 (0.6%)	

ALL, acute lymphoblastic anemia; AML, acute myeloid leukemia; MDS, myelodysplastic syndrome; Mel, melphalan; Cy, cyclophosphamide; VP16, etoposide.

*Bartelink* et al. reported the results of a retrospective study of 102 pediatric patients (median age 3.1 years [range 0.2–21.0)] undergoing allogeneic HSCT for malignant (45%) and non-malignant (55%) indications. Patients received conditioning with busulfan, cyclophosphamide and melphalan (BuCyMel) (43%) or in other combinations. A once daily regimen was given in 63% of the patients, the rest received Bu 4 times daily. OS, EFS and toxicity were associated with Bu exposure. In multivariate analysis, a cumulative Bu exposure between 72 and 80 mg*h/L was associated with the most favorable EFS and OS. Higher AUC was associated with a lower incidence of graft failure and relapse. Higher Bu exposure was also a significant predictor for aGVHD, but not for veno-occlusive disease (VOD) or mucositis (Bartelink et al., 2009).

*Ansari* et al. performed a prospective study to examine the association between i. v. Bu exposure and clinical outcome in a pediatric cohort of 75 patients [median age 3.2 years (range 0.1–20.0)]. Patients were included with malignant (64%) and non-malignant diseases (36%). The majority of patients received a conditioning regimen consisting of BuCy (89%) and Bu was given 4 times daily over 4 days. They found that an average Bu concentration of the first dose (C_ss,day1_) > 600 ng/ml (corresponding with a daily AUC of 14.4 mg*h/L or cumulative AUC of 57.6 mg*h/L) was associated with higher incidence of aGvHD and higher risk of non-relapse mortality (NRM). In multivariate analysis, C_ss,day1_ > 600 ng/ml was associated with lower EFS and lower OS (Ansari et al., 2014).

A landmark study done by *Bartelink* and others in 2016 included 674 patients [median age 4.5 years (range 0.1–30.4)] from 15 different pediatric transplantation centers. Malignant (41%) and non-malignant (59%) indications were included and the majority received a conditioning regimen with BuCy (52%), followed by BuFlu (37%) and BuCyMel (10%). The main outcome of interest was EFS; secondary outcomes were graft failure, relapse, TRM, acute toxicity, cGvHD, OS and cGvHD free survival. They defined that a target of 90 mg*h/L (range 78–101 mg*h/L) gave the highest probability of EFS. Compared with the low AUC group (<78 mg*h/L), the optimal AUC decreased the probability of graft failure or disease relapse and a high AUC (>101 mg*h/L) increased the risk of TRM and acute toxicities (Bartelink et al., 2016).

*Benadiba* et al. conducted a study with 36 pediatric patients [median age 5.9 years (range 0.6–19.3)] receiving a umbilical cord blood (UCB) transplantation for a myeloid malignancy. All patients received Bu in a regime of 4 times daily in combination with Cy (91.7%), Mel (6%) or Cy plus etoposide (2.3%). In multivariate analysis, C_ss,day1_ > 600 ng/ml (daily AUC of 14.4 mg*h/L or cumulative AUC of 57.6 mg*h/L) was a significant risk factor for OS and EFS. Furthermore, neutrophil and platelet recovery and non-relapse mortality were significantly higher in patients with C_ss,day1_ < 600 ng/ml than C_ss,day1_ > 600 ng/ml (Benadiba et al., 2018).

*Philippe* et al. specifically looked at the occurrence of VOD in relationship with Bu exposure. In this retrospective study, 293 pediatric patients with a median age of 6.2 years (0.2–21) were included of whom 75 (25.6%) developed VOD. There was a 6-fold increased risk of VOD in patients with a maximum drug concentration level (C_max_) of ≥1.88 ng/ml. Also, weight <9 kg and age <3 years were independent predictors of VOD (Philippe et al., 2019).

Together, these data suggest that overexposure to Bu (either on day one, or overall AUC) has a negative effect on OS and EFS. A cumulative AUC of 78–101 mg*h/L or C_ss,day1_ < 600 ng/ml are suggested as possible targets. A target value for the first dose below 600 ng/ml (= 14.4 mg*h/L per day and 57.6 mg*h/L in total) seems rather low, but adequate overall exposure over the course of the treatment could still be achieved because of decreased clearance of Bu over time (Gaziev et al., 2010; Bartelink et al., 2012; Kawazoe et al., 2018). On the other hand, the target suggested by Bartelink et al. is higher than the historical target of 56–86 mg*h/L (Css 600–900 ng/ml), which seems to be in contrast with the results of Ansari et al. Also, the study done by Bartelink et al. shows that low cAUC (<78 mg*h/L) gave a higher risk of graft failure or disease relapse. However, the considerable variability in Bu dosing (once, twice or four times daily), difference in exposure targets (C_ss_, cAUC, AUC_dose_), difference of exposure units (mg*h/L, µM*min), the method of exposure estimation and co-medication (cyclophosphamide versus fludarabine) makes comparison of all these results difficult and complex. Also, optimal exposure may differ between groups based on factors, such as underlying disease, age and comorbidities (McCune et al., 2000). A proposal of harmonizing Bu exposure unit to mg*h/L has been done and will hopefully lead to more accurate assessment of exposure and thereby evaluation of outcomes in multicenter studies (McCune et al., 2019).

### Treosulfan

In the last decade, treosulfan (Treo) has gained popularity as a chemotherapeutic agent in conditioning regimens prior to HSCT for malignant and non-malignant disorders. It is a water-soluble bifunctional alkylating agent and a structural analogue of busulfan. Although Treo has structural similarities with Bu, its mechanism of alkylation is different. As a pro-drug, it undergoes non-enzymatic and pH-dependent conversion into active mono- and diepoxide derivatives under physiological conditions. These derivatives cause DNA alkylation and interstrand DNA crosslinking, leading to DNA fragmentation and apoptosis (Hartley et al., 1999). Approximately 25–40% of Treo is excreted renally in unchanged form (Hilger et al., 1998). Interpatient variability of clearance in children is high; between 30 and 68% have been reported in population pharmacokinetic studies (Mohanan et al., 2018; van der Stoep et al., 2019; Chiesa et al., 2020). Age, bodyweight and renal clearance are covariates that were found to (partially) explain the large interindividual variability. More recently, the relationship between Treo exposure and clinical outcome has been explored in several studies with pediatric patients undergoing HSCT. Table 2 summarizes the reports of Treo PK associated with outcome in pediatric patients.

**TABLE 2:** Reported associations of pharmacokinetic parameters of treosulfan and clinical outcomes.

First author, year	*n*	Age, median (range)	Diagnosis	Regimen	Dose	Major findings
van der Stoep et al. (2017)	77	4.8 (0.2–18.3)	HBP: 31 (40.3%)	TreoFlu: 25 (35.5%)	3 × 10 g/m^2^: 12 (15.6%)	High Treo AUC_0-∞_ (>1,650 mg*h/L per day) was associated with a higher risk of ≥ grade 2 mucositis (OR 7.03; 95%CI 1.60–30.86, *p* = 0.01). There is also an increased risk of skin toxicity (OR 9.96; 95%CI 1.85–53.46, *p* = 0.007).
			Hem. malig: 12 (15.6%)	TreoFluThio: 52 (67.5%)	3 × 14 g/m^2^: 65 (84.4%)	
			IEI: 22 (28.5%)			
			BMF: 11 (14.3%)			
			Other: 1 (1.3%)			
Mohanan et al. (2018)	87	9.0 (1.5–25)	TM: 87	TreoFluThio	3 × 14 g/m^2^: 87 (100%)	In a *post-hoc* analysis, lower Treo clearance (<7.97 L/h/m^2^) was associated with poor overall survival (HR 2.7; 95%CI 1.09–6.76, *p* = 0.03) and event free survival (HR 2.4; 95%CI 0.98–5.73, *p* = 0.055). No association with toxicity.
Chiesa et al. (2020)	87	1.6 (0.2–16.7)	IEI: 79 (91%)	TreoFlu	3 × 10 g/m^2^: 4 (5%)	Higher cumulative Treo AUC_0-∞_ showed higher risk of mortality in multivariable analysis (HR 1.32; 95%CI 1.07–1.64, *p* = 0.0093), a trend was seen for low AUC_0-∞_ associated with poor engraftment (HR 0.61; 95%CI 0.36–1.04, *p* = 0.072) in univariable analysis. TRM was higher in patients with AUC>6,000 mg*h/L than <6,000 mg*h/L (39% vs. 3%, *p* = 0.00001). A cumulative AUC_0-∞_ of 4,800 mg*h/L is proposed as target.
			IBD: 5 (5%)		3 × 12 g/m^2^: 23 (26%)	
			JMML: 2 (2%)		3 × 14 g/m^2^: 60 (69%)	
			IEM: 1 (1%)			
van der Stoep et al. (2021)	110	5.2 (0.2–18.8)	IEI: 38 (35%)	TreoFlu: 37 (32%)	3 × 10 g/m^2^: 18 (16%)	All grade mucositis was associated with high Treo AUC_0-∞_ (OR 4.43; 95%CI 1.43–15.50, *p* = 0.01), but not mucositis ≥2 or higher (OR 1.51; 95%CI 0.52–4.58, *p* = 0.46). Skin toxicity ≥ grade 2 was associated with high AUC_0-∞_ (OR 3.97; 95%CI 1.26–13.67, *p* = 0.02). No association with 1-year donor chimerism, 2-years OS and EFS.
			HBP: 55 (50%)	TreoFluThio: 77 (68%)	3 × 14 g/m^2^: 92 (84%)	
			BMF: 17 (15%)			

HPB, hemoglobinopathies; hem. malig, hematological malignancies; IEI, inborn errors of immunity; BMF, bone marrow failure; TM, thalassemia major; IBD, inflammatory bowel disorder; JMML, juvenile myelomonocytic leukemia; IEM, inborn errors of metabolism.

*Van der Stoep* et al. described a pediatric cohort of 77 patients transplanted for non-malignant (84.4%) and malignant (15.6%) diseases [median age 4.8 years (range 0.2–18.3)]. Patients received Treo with fludarabine only (35.5%) or with additional thiotepa (67.5%). Twelve patients <1 year of age received a total dose of 30 g/m^2^ and 65 patients ≥1 year of age received 42 g/m^2^. Patients were divided into three exposure groups (on day 1); low (<1,350 mg*h/L, medium (1,350–1,650 mg*h/L) and high (>1,650 mg*h/L). Patients in the high exposure group had an higher risk for mucosal and skin toxicity compared to the low exposure group. The risk of experiencing two or more toxicities was also higher in the high exposure group compared with the low exposure group. No relationship was found between exposure and aGvHD, engraftment, chimerism and survival (van der Stoep et al., 2017).

In a study done by Mohanan et al., 87 patients with thalassemia major undergoing HSCT were included to study the PK of Treo in relationship with outcome. The majority of included patients were children, although some adults up to 25 years of age were also included [median age 9.0 years (range 1.5–25)]. Treo was given in combination with fludarabine and thiotepa in a total dose of 42 g/m^2^. The influence of Treo PK on rejection, toxicities, OS, EFS and TRM was evaluated and no association was found with these outcome parameters. A trend was seen towards better OS with high Treo clearance (>7.97 L/h/m2) and low day 1 AUC (<1828 mg*h/L). In a *post-hoc* analysis they found that lower Treo clearance (<7.97 L/h/m^2^) was significantly associated with poor OS and EFS (Mohanan et al., 2018).

Chiesa et al. investigated the relationship between Treo PK and OS and donor engraftment in 87 children [median age 1.6 years (range 0.2–16.7)], transplanted mainly for an inborn error of immunity (91%). All patients received Treo with fludarabine with a total dose of 42 g/m^2^ in children aged >12 months, 36 g/m^2^ in children aged 3–12 months and 30 g/m^2^ in children ≤3 months. A higher Treo cumulative AUC (the sum of Treo AUC on 3 days, cAUC) showed a higher risk of mortality in multivariable analysis. Also, children with cAUC >6,000 mg*h/L had higher TRM than children with cAUC <6,000 mg*h/L (39% vs. 3%). A trend was seen for low AUC to be associated with poor donor engraftment (≤20%), but this was observed only in univariable analysis. The authors propose a therapeutic target of cAUC 4,800 mg*h/L, corresponding with 1,600 mg*h/L daily (Chiesa et al., 2020).

Very recently, *Van der Stoep* et al. published results on Treo PK in a cohort of 110 pediatric patients with non-malignant diseases [median age 5.2 years (range 0.2–18.8)]. The influence of Treo PK on early and long-term clinical outcome was evaluated. The main outcome of interest was 2-years EFS and secondary outcomes were 2-years OS, toxicities, engraftment, donor chimerism and GvHD. No association was found between Treo PK and 2-years EFS, nor with 2-years OS, engraftment, donor chimerism and GvHD. High Treo exposure (>1750 mg*h/L) on day 1 was associated with all grade mucositis, but not with mucositis ≥ grade 2. High Treo exposure was also associated with ≥ grade 2 skin toxicity (van der Stoep et al., 2021).

While there seems to be a relationship with Treo PK and mucositis in the first study of *Van der Stoep* et al., this was not confirmed by *Mohanan* and *Chiesa* et al. Furthermore, in a more recent study of *Van der Stoep* et al., only a relationship between exposure and all grade mucositis was seen, but not with grade 2 or higher, which is clinically more relevant. However, in both studies of *Van der Stoep* et al. as well as the study of *Chiesa* et al., high Treo exposure was related to the risk of ≥ grade 2 skin toxicity. In terms of survival, *Chiesa* et al. showed a relationship between exposure and OS, while *Mohanan* et al. hinted towards a trend and *Van der Stoep* et al. did not observe a relationship. These differences could possibly be explained by interindividual variability in exposure between the studies, which was higher in the studies of *Chiesa* et al. and *Mohanan* et al. Also, no relationship with EFS was found (Mohanan et al., 2018; van der Stoep et al., 2021) and overall, it is noticed that Treo is well tolerated, with limited regimen-related toxicities, while still achieving good results when it comes to clinical outcome. Together, these results indicate a moderate exposure-toxicity relationship, but a relationship with survival is not evident and consistent. The clinical value of TDM could be investigated to prevent skin toxicity, although implementation of preventive care guidelines could possibly reduce the incidence of cutaneous complications as well. The current evidence do not justify the use of TDM in routine patient care, but can be useful in specific cases and subgroups and warrants further investigation.

### Fludarabine

The purine analogue fludarabine (Flu) has become an alternative for cyclophosphamide (Cy) in the classical myeloablative conditioning regimen Bu-Cy, because of the lower risk NRM without compromising efficacy (Ben-Barouch et al., 2016). Flu is currently being used as part of various different conditioning regimens, whether it be myeloablative, reduced-intensity or non-myeloablative. Fludarabine phosphate is a prodrug that is rapidly converted into F-ara-A in the systemic circulation. Subsequently, F-ara-A is phosphorylated in the cell into the active metabolite fludarabine triphosphate, F-ara-ATP, which is responsible for the inhibition of DNA synthesis and RNA production, leading to apoptosis (Gandhi and Plunkett 2002). Flu is predominantly excreted renally. Interpatient variability in clearance is high and bodyweight and renal clearance were found to be contributing factors to this variability (Ivaturi et al., 2017; Chung et al., 2018; Langenhorst et al., 2018). Table 3 summarizes the reports of Flu PK associated with outcome in pediatric patients.

**TABLE 3:** Reported associations of pharmacokinetic parameters of fludarabine and clinical outcomes.

First author, year	*n*	Age, median (range)	Diagnosis	Regimen	Dose	Major findings
Ivaturi et al. (2017)	133	5.0 (0.2–17.9)	Hem. malig: 59 (44%)	BuFlu: 40 (30%)	3–5 x 40 mg/m^2^: 55 (41%)	No association with Flu and TRM (*p* = 0.35). In the malignancy group DFS was highest at 1 year post HSCT in patients with a cumulative AUC >15 mg*h/L compared to <15 mg*h/L (82.6 vs. 52.8%, *p* = 0.04). A cumulative AUC of >15 mg*h/L is considered as a minimum exposure threshold.
			IEI: 18 (14%)	FluCy: 45 (34%)	3–5 x 12.5–35 mg/m^2^: 40 (30%)	
			HBP: 8 (6%)	BuFluClo: 18 (14%)	3–5 x 0.9–1.22 mg/m^2^: 38 (29%)	
			Metabolic: 22 (16%)	FluThioMel: 15 (11%)		
			BMF: 22 (16%)	Other: 15 (11%)		
			Epidermolysis bullosa: 4 (4%)			
Mohanan et al. (2017)	53 (no. of children not specified)	17 (3–57)	AA: 40 (75%)	FluCy: 29 (55%)	6 × 30 mg/m^2^	AUC >29.4 µM*h was a significant factor associated with aGVHD in multivariate analysis (*p* = 0.02)
			FA: 13 (25%)	FluCyTBI: 20 (38%)		None of the PK parameters showed any association with engraftment, mixed chimerism, rejection, overall survival or TRM.
				FluCyATG: 4 (7%)		
Chung et al. (2018)	43	11.8 (1.3–18.5)	Acute leukemia: 29 (67.4%)	BuFluVP16: 24 (55.8%)	6 × 40 mg/m^2^: 40 (93%)	No significant association was found between AUC and toxicities, GvHD, relapse and survival.
			Other malig: 2 (4.7%)	BluFlu: 12 (27.9%)	5 × 40 mg/m^2^: 3 (7%)	
			Non-malignant: 12 (28%)	BuFluMel: 4 (9.3%)		
				FluCy: 2 (4.7%)		
				BuFluCy: 1 (2.3%)		
Langenhorst et al. (2019)	192 (119 adults, 73 children	36.2 (0.23–74)	Benign: 68 (35%)	BuFlu	4 × 40 mg/m^2^	Flu exposure is a predictor for EFS. NRM was increased with high Flu exposure (*p* < 0.001) and more graft failure was seen with low exposure (*p* = 0.04). An optimal cumulative AUC of 20 mg*h/L (±5) is suggested. The optimal exposure group had a significantly higher EFS compared with the above-optimal exposure group (HR 2.0; 95%CI 1.1–3.5, *p* = 0.01) and (non-significantly) higher than the below-optimal group (HR 1.8; 95%CI 0.72–4.5, *p* = 0.21).
			Leukemia/lymphoma: 71 (37%)			
			MDS: 30 (16%)			
			Plasma cell disorder: 23 (12%)			

AA, aplastic anemia; FA, fanconi anemia; TBI, total body irradiation.

*Ivaturi* et al. reported a prospective PK study of 133 pediatric patients transplanted for malignant (44%) and non-malignant (56%) indications [median age 5.0 years (range 0.2–17.9)]. Patients received Flu in various different conditioning regimens and in different dosages. No association was found between Flu exposure and the primary endpoint TRM. The highest 1-year OS rate was seen in patients with a cumulative AUC (cAUC) between 15 and 19 mg*h/L, however this was not statistically significant. In the malignant subgroup, 1- year disease free survival (DFS) was higher in patients with a cAUC between 15 and 19 mg*h/L than <15 mg*h/L (82.6% vs. 52.8%). Based on the data in their study, the authors propose a minimum exposure threshold of 15 mg*h/L to achieve the best possible outcome (Ivaturi et al., 2017).

*Mohanan* et al. studied the pharmacokinetics of Flu in 53 patients with aplastic anemia (75%) and Fanconi anemia (25%). They included both children and adults, however the number of children was not specified [median age 17 years (range 3–57)]. The majority of patients received a regimen with Flu and Cy (55%), others received Flu and Cy in combination with TBI (38%) or anti-thymocyte globulin (ATG) (7%). All patients received a dose of 30 mg/m^2^ daily for 6 days. There was no association between the PK parameters of Flu and engraftment, mixed chimerism, rejection, OS or TRM. In multivariate analysis, a cAUC of >29.4 µM*h was associated with a higher risk of aGVHD (Mohanan et al., 2017).

Chung et al. described the pharmacokinetics of Flu in 43 Korean pediatric patients [median age 11.8 years (range 1.3–18.5)]. The majority of patients received a transplantation for a malignant disease (72.1%). Flu was given in combination with various different agents, but the majority received a regimen with Bu and etoposide (55.8%) with a daily dose of 40 mg/m^2^ for 6 days. In their exploratory analyses, they did not find any relationship between Flu cAUC and toxicities, GVHD, relapse, EFS and survival (Chung et al., 2018).

The most recent study is from *Langenhorst* and others, who conducted a retrospective cohort analysis in 192 patients [119 adults and 73 children, median age 36.2 years (range 0.23–74)]. All patients received a conditioning regimen of BuFlu (4 × 40 mg/m^2^), mostly for malignant diseases (65%). They found an increased incidence of NRM with higher Flu cAUC and more graft failures were observed with lower Flu cAUC. No influence on relapse was seen. Based on these results, they calculated that a cAUC of 15–25 mg*h/L was the optimal target window for Flu to minimize the chance of an event. When considering three exposure groups (below-optimal, optimal and above-optimal), the optimal exposure group had a significantly higher EFS compared with the above-optimal exposure group and (non-significantly) higher than the below-optimal group. NRM was the main cause of an event in the above-optimal group and immune reconstitution was significantly lower, whereas the risk of graft failure and NRM was increased in the below-optimal group (Langenhorst et al., 2019).

The abovementioned studies show variable results. *Langenhorst* et al. showed that Flu exposure within the optimal target (cumulative AUC of 15–25 mg*h/L) had significant higher EFS than the above-optimal group and *Ivaturi* et al. showed better DFS with a cumulative Flu exposure >15 mg*h/L in a subgroup of 59 children with malignancy. However, Mohanan et al. and Chung et al. failed to show associations with EFS and OS. Patient cohorts in the last two studies were small (53 and 43, respectively), so it is possible that a statistically significant relationship could not be detected. Also, in all studies except *Langenhorst* et al. various different conditioning regimens were included with various Flu dosage schemes, which makes comparison of the results difficult. Currently, a randomized phase II study is ongoing to study the influence of individualized fludarabine conditioning on the incidence of severe viral infections and other transplant-related outcomes in adult patients with hematological malignancies (Clinicaltrialsregister.eu: TARGET study 2018-000356-18)). Whether these results can be extrapolated to children remains to be determined. Ideally, a randomized study in children is done to address whether individualized dosing improves clinical outcome. For now, the evidence for TDM for Flu is growing, but more studies are needed to explore whether a single optimal target can be defined. In the meantime, the use of TDM in routine patient care remains limited.

### Clofarabine

The addition of clofarabine (Clo) to the conditioning regimen with Bu and Flu prior to HSCT in pediatric hematological malignancies has proven to be a safe and promising strategy (Alatrash et al., 2016; Versluys et al., 2021). Similar as fludarabine, clofarabine is a purine analogue and a prodrug that is converted intracellularly to its active metabolite clofarabine-5’-triphosphate. This metabolite inhibits DNA polymerase-α, resulting in inhibition of DNA synthesis and repair. Furthermore, it disrupts mitochondrial membrane integrity, leading to apoptosis (Bonate et al., 2006). Excretion is predominantly through the kidneys. Very recently, the pharmacokinetics of Clo in pediatric HSCT recipients have been characterized by two groups (Wang et al., 2019; Nijstad et al., 2021). Bodyweight, age and renal function were covariates influencing clofarabine variability in clearance. Exposure-response relationships between clofarabine and clinical outcome have not been published so far.

### Thiotepa

Thiotepa is an alkylating drug that is often combined with Treo and Flu or Bu and Flu in a myeloablative regimen. It is given in a dose of 8–10 mg/kg, (usually 8 mg/kg once or 5 mg/kg for 2 days). Because of its highly lipophilic nature and therefore its ability to cross the blood brain barrier, the addition of thiotepa not only adds myeloablative ability, but may also be beneficial in diseases with central nervous system involvement (Naik et al., 2020). Thiotepa is quickly metabolized in the liver into the active metabolite triethylene phophoramide (TEPA), which has a comparable alkylating activity as thiotepa. By cross-linking of DNA strands, these compounds inhibit DNA, RNA and protein synthesis. Thiotepa and TEPA are eliminated in urine, but also dermally via sweat (Horn et al., 1989). The pharmacokinetics of thiotepa has been studied in adults and children, but not in the allogeneic HSCT setting (Heideman et al., 1989; Huitema et al., 2001; Kondo et al., 2019).

## Serotherapy

### ATG

Serotherapy with rabbit anti-thymocyte globulin (ATG) or anti-T lymphocyte globulin (ATLG) is often added to the conditioning regimen in pediatric allogeneic HSCT for prophylaxis against GvHD and graft rejection. ATG is a rabbit polyclonal IgG that is produced by the immunization of rabbits with human thymocytes (Thymoglobulin®, Sanofi Genzyme), whereas ATLG is generated upon immunization with the Jurkat T-cell line (Grafalon®, Neovii Pharmaceuticals AG). Both ATG and ATLG contain antibodies recognizing antigens expressed on the surface of many immune and non-immune cells, and several mechanisms by which ATG/ATLG eliminates these targeted cells are described, including inducing apoptosis, complement-dependent lysis or NK-cell mediated lysis (Mohty 2007). Due to the differences in the manufacturing of both products, the lymphodepleting capacity of both brands is not the same. This is reflected in the total dosage given, which varies in the pediatric setting for ATG between 4.5 and 10 mg/kg while for ATLG it is much higher (15–45 mg/kg). The fraction that is capable of lymphocyte binding is also described as active ATG/ATLG and is only a minor part of the total rabbit IgG (total ATG/ATLG) dosage. The lympholytic level of active ATG/ATLG is 1 AU/mL (Waller et al., 2003). ATG/ATLG is given i.v. and the total dosage is often divided over 3–4 days. As for all antibodies, target binding is besides the main mechanism of action also one of the main clearance mechanisms of ATG/ATLG together with non-specific degradation. A third clearance method, leading to rapid elimination of ATG/ATLG, may occur when anti-drug-antibodies (anti-ATG/ATLG) are developed (Jol-van der Zijde et al., 2012). The pharmacokinetics and–dynamics (PD) of ATG in the pediatric HSCT setting have been described, however only in a limited number of studies (Call et al., 2009; Admiraal et al., 2015a; Admiraal et al., 2015b; Admiraal et al., 2016). Interindividual variability for linear clearance is reported to be between 50% and 86%, with body weight and absolute lymphocytes number pre-ATG as important covariates (Call et al., 2009; Admiraal et al., 2015b). For ATLG, no population PK models have been published so far, and knowledge about its PK and PD is only obtained from a few studies investigating concentration-time curves (Oostenbrink et al., 2019; Vogelsang et al., 2020). Table 4 summarizes the reports of ATG PK associated with outcome in pediatric patients.

**TABLE 4:** Reported associations of pharmacokinetic parameters of ATG and clinical outcomes.

First author, year	*n*	Age, median (range)	Diagnosis	Regimen	Dose ATG	Major findings
Call et al. (2009)	13	10 (2–16)	AML: 4 (31%)	TBI/Thio/CY	Thymoglobulin	Weight-based dosing regimen (total dose 10 mg/kg) of Thymoglobulin was effective and well tolerated by all patients. None of the patients developed grade III-IV aGvHD.
			ALL: 3 (23%)		10 mg/kg, administered as 1 mg/kg on day -4 and 3 mg/kg/day on days -3 to −1	
			CML: 3 (23%)			
			JCML: 2 (15%)			
			MDS: 1 (8%)			
Admiraal et al. (2015a)	251	6.2 (0.2–22.7)	Malignancy: 116 (46%)	RIC	Thymoglobulin	Individualized dosing of ATG could result in improved outcomes. For the CB group, AUC ≥20 AU × day/mL decreased immune reconstitution in CB, but decreased immune reconstitution was noted only if AUC ≥100 AU × day/mL in BM and PB. Successful immune reconstitution by day 100 was associated with increased OS. An AUC before HSCT of ≥40 AU × day/mL resulted in a lower incidence of aGvHD, cGvHD and graft failure compared with an AUC <40 AU × day/mL.
			IEI: 51 (20%)	MAC – chemo	<9 mg/kg 4%	
			BMF:15 (6%)	MAC - TBI	9–11 mg/kg 94%	
			Non-malignant: 69 (27%)		>11 mg/kg 2%	
					Day start ATG -5, dose divided over 4 days	
Admiraal et al. (2016) ^a^	137	7.4 (0.2–22.7)	ALL: 22 (16%)	Bu-Flu	Thymoglobulin	Low ATG exposure (AUC <16 AU^a^day/mL) was the best predictor for CD + T cell recovery in CB transplant. Patients with a high AUC had a significantly lower EFS compared to low exposure or without ATG. Every 10-point increase in ATG exposure resulted in 5% lower survival probability. Patients receiving ATG had a significantly lower incidence of aGvHD (III-IV) compared with those not receiving ATG (HR, 0.27; 95% CI, 0.08–0.86; *p* = 0.027).
			AML: 30 (22%)	Bu-Flu-Clo	Before 2010	
			Lymphoma: 4 (3%)	TBI based	10 mg/kg	
			IEI: 33 (24%)	Cy-Flu	Day start ATG -5, dose divided over 4 days	
			BMF: 7 (5%)		After 2010	
			Benign non-IEI (41 (30%)		<40 kg: 10 mg/kg	
					>40 kg: 7.5 mg/kg	
					Day start ATG -9, dose divided over 4 days	
Oostenbrink et al. (2019)	58	9 (1–18)	ALL: 33 (57%)	Chemo + TBI	Thymoglobulin	Active ATG of both ATG products was cleared at different rates, more variability in the Thymoglobulin treated group. Patients treated with Grafalon had a median level of 27.9 AU/mL and with Thymoglobulin 10.6 AU/mL at day 0. Three weeks after HSCT, 15/16 Grafalon patients had an active ATG level <1 AU/mL while 17/42 Thymoglobulin patients had still active ATG levels above this threshold. For Thymoglobulin, exposure to ATG was significantly higher with 10 mg/kg compared to 6–8 mg/kg and was associated with delayed immune recovery. Occurrence of aGvHD (grade III–IV) was highest in the Thymoglobulin low dosage group.
	42 Thymoglobulin	6 (1–17)	AML: 25 (43%)	Chemo	8.7 (6.0–10.5) mg/kg	
	16 Grafalon				Grafalon	
					53 (45–60) mg/kg	
Vogelsang et al. (2020)	32	5.3 (0.1–17.3)	Non-malignant: 22 (69%)	TreoFluThio	Thymoglobulin	Grafalon and Thymoglobulin show different pharmacological and immunological impact in children. Active plasma levels for Grafalon were less variable compared to Thymoglobulin. Median active peak plasma levels were 77.9 μg/ml for Grafalon and 8.11 μg/ml for Thymoglobulin. Incidence of GvHD was similar for patients with high (above the median) or low (below the median) exposure. Immune recovery of total leucocytes and T cells was delayed in patients with high ATG exposure. No significant difference was found for overall survival.
	22 Thymoglobulin	13.7 (1.5–17.2)	Malignant: 10 (31%)	NMA	4.5–10 mg/kg	
	10 Grafalon			TBI/VP-16	Grafalon	
					30–60 mg/kg	

a 66 patients (48%) were included in the previous analysis of 2015. (J)CML: (juvenile) chronic myeloid leukemia, RIC, reduced intensity conditioning; MAC, myeloablative conditioning.

*Call* et al. evaluated the pharmacokinetics of total and active ATG Thymoglobulin in a prospective trial with 13 children [median age 10 years (range 2–16)] who underwent an unrelated donor HSCT with non-T-cell–depleted bone marrow grafts for hematologic malignancies. There were no occurrences of grade III–IV acute GvHD and none of the patients had serious infections following transplantation. Call et al. concluded that the use of a 10 mg/kg dose of ATG in children with hematologic malignancies can be administered without increasing the risk of rejection, or serious infection in pediatric patients with a low rate of GvHD (Call et al., 2009).

*Admiraal* et al. described in 2015 the pharmacokinetics of ATG in a much larger patient cohort, including 267 HSCT patients from two study centers (Admiraal et al., 2015b). With the use of a population PK model, pharmacokinetic endpoints (i.e., AUC) were calculated and studied in relation to the clinical outcome measures of the patients, to determine the therapeutic window and the optimal active Thymoglobulin exposure. The results of this analysis were published in a separate publication. Successful immune reconstitution, defined as CD4^+^ T cells >0.05 × 10^9^ cells/L within 100 days, was lower in patients with a higher AUC post-HSCT (for patients receiving a cord blood graft ≥20 AU x day/mL, and for patients with a bone marrow or peripheral blood stem cell graft ≥100 AU x day/mL) and correlated with TRM and viral reactivations. A lower risk for graft failure and acute GvHD was seen in patients with an AUC pre-HSCT of ≥40 AU x day/mL compared to patients with an AUC less than 40 AU x day/mL (Admiraal et al., 2015a).

Based on these two publications, *Admiraal* et al. developed an individualized dosing regimen taken body weight, baseline lymphocytes pre-ATG and stem cell source for each patient into account. The effectiveness of this individualized dosing regimen was assessed in a cohort of 137 children receiving a cord blood graft and in a prospective, open-label, phase II clinical trial including 58 patients and 112 historical controls. Chance of successful immune recovery was significantly increased in the individualized dosing group in both studies, but no differences were seen between patients with low or high ATG exposure for severe acute GvHD (grade III-IV) and failure of the graft (Admiraal et al., 2016; Admiraal et al., 2022).

Concluding from the above-mentioned publications, using an individualized dosing regimen for ATG could improve patient outcome. Both ATG population PK models described so far showed large interpatient variability, which could be minimized by applying TDM. However, TDM for ATG at this moment is time-consuming, expensive and the assays to measure active ATG are to our knowledge performed only at a few centers worldwide. For ATLG, both studies assessing the PK/PD mentioned differences in the pharmacological and immunological impact between ATLG and ATG (Oostenbrink et al., 2019; Vogelsang et al., 2020). The next step would be to assess whether there is a relationship between ATLG drug concentrations and clinical and immunological outcome in order to determine if TDM could be useful.

### Alemtuzumab

Besides ATG/ATLG, an alternative lymphodepleting drug that is often used as serotherapy is Alemtuzumab (Campath®). Alemtuzumab is a humanized monoclonal antibody targeting CD52, which is expressed on the surface of various hematopoietic cells. Alemtuzumab can be given subcutaneously or intravenously for *in vivo* depletion of immune cells, but the use of alemtuzumab for *in vitro* T-cell depletion, by adding alemtuzumab to the graft before infusion, has also been described (Barge et al., 2006; von dem Borne et al., 2006). The total dose given in children usually varies between 0.5 and 1.5 mg/kg, however for some diseases (such as hemophagocytic lymphohistiocytosis (HLH)) much higher dosages are being used. The lytic level of alemtuzumab in humans is presumed to be near 0.1–0.16 μg/ml (Marsh et al., 2016; Riechmann et al., 1988). Based on the few studies analyzing alemtuzumab PK and PD in the pediatric HSCT setting (see for an overview Table 5), a difference between ATG and alemtuzumab PK is clearance, both linear and saturable, which is lower for alemtuzumab. Furthermore, the interindividual variability for alemtuzumab clearance is described to be much higher than for ATG (Admiraal et al., 2019).

**TABLE 5:** Reported associations of pharmacokinetic parameters of alemtuzumab and clinical outcomes.

First author, year	*n*	Age, median (range)	Diagnosis	Regimen	Dose alemtuzumab	Major findings
Marsh et al. (2016)	105	4.7 (0.3–27.2)	HLH: 54 (51%)	FluMel	Distal dosing	Peritransplant alemtuzumab levels have impact on the incidence of aGvHD, mixed chimerism and lymphocyte recovery. 18% developed GvHD with alemtuzumab levels ≥0.16 μg/ml, 68% in patients with levels ≤0.15 μg/ml. Mixed chimerism occurred in 21% of the patients with ≤0.15 μg/ml, in 42% with levels between 0.16 and 4.35 μg/ml and in 100% if levels were above 4.35 μg/ml. Patients with levels ≥0.57 μg/ml had lower T-cell counts at day 100. A therapeutic range at day 0 of 0.2–0.4 μg/ml is recommended.
			BMF: 13 (12%)		3/10/15/20 mg over days -22 to −19	
			(S)CID: 17 (17%)		<10 kg: 3/10/10/10 mg	
			CGD: 5 (5%)		Intermediate dosing	
			Metabolic: 4 (4%)		1 mg/kg over days -14 to −10	
			SCD: 2 (2%)		Proximal dosing	
			Other: 10 (10%)		3/10/15/20 mg or 1 mg/kg starting at day -12 or closer to HSCT	
Bhoopalan et al. (2020)	13	15.5 (3–21)	ALL: 8 (61.5%)	FluThioMelRitux	Subcutaneous n = 8	BSA-based dosing of alemtuzumab is feasible in pediatric haplo-transplantation patients. AUC of alemtuzumab did not have a significant relation with OS, engraftment, IR and GvHD.
			AML: 3 (23.1%)		Subcutaneous and intravenous n = 5	
			CML: 1 (7.7%)		Test dose of 2 mg/m^2^ plus total dose of 45 mg/m^2^	
			Therapy-related MDS: 1 (7.7%)		Dose given from days -14 to -11	
Dong et al. (2021)	29	6.4 (0.28–21.4)	HLH: 13 (45%)	FluMel	Subcutaneous	Proposed therapeutic range of 0.15–0.6 μg/ml on the day of transplantation is associated with better HSCT outcomes (less aGVHD and improved lymphocyte recovery). To achieve this optimal level allometric or BSA-based dosing is advised. Top-up dose on day -3 for patients who, based on individualized PK estimation, will have a concentration <0.15 μg/ml on the day of transplantation is recommended.
			CGD: 2 (7%)		0.5–0.6 mg/kg	
			IPEX: 2 (7%)		1 mg/kg	
			SAA: 5 (17%)		Dose given days -14 to −10 or -14 – -12	
			(S)CID: 3 (10%)		Top-up dose was given either on day -3 or -1	
			Other: 4 (14%)			

HLH, hemophagocytic lymphohistiocytosis; (S)CID, (severe) combined immune deficiency; CGD, chronic granulomatous disease; SCD, sickle cell disease; IPEX, immunodysregulation polyendocrinopathy enteropathy X-linked syndrome; SAA, severe aplastic anemia; Ritux, rituximab.

In 2016 *Marsh* et al. reported their recommended therapeutic range of alemtuzumab at the day of transplantation of 0.2–0.4 μg/ml. They investigated the relation between alemtuzumab concentrations at day HSCT with several clinical outcome parameters in 105 (mainly) pediatric patients [median age 4.7 years (range 0.3–27.2)]. A level ≤0.15 μg/ml at the day of transplantation was associated with a lower incidence of mixed chimerism, however also led to a higher probability of acute GvHD. For T-cell recovery at day 100 after transplantation, day 0 alemtuzumab levels ≥0.57 μg/ml were correlated with lower T-cell counts (Marsh et al., 2016).

*Bhoopalan* et al. described the pharmacokinetics of alemtuzumab in 13 patients [median age 15.5 years (range 3–21)] with haploidentical HSCT. Alemtuzumab was given subcutaneous from days -14 to -11 using a BSA-based dosing, except for five patients who received intravenous dosing for their last two doses. Patients received a test dose of 2 mg on day -4 followed by a total dose of 45 mg/m^2^ in escalating doses of 10, 15 and 20 mg/m^2^ on days -13, -12 and -11. Ten of 13 patients had detectable alemtuzumab levels at week 4 after HSCT. Median AUC was 117.1 (range 28.1–165.4) µg*day/mL. No significant correlation was found between AUC and clinical outcome parameters such as overall survival, engraftment, lymphocyte counts and GvHD (Bhoopalan et al., 2020).

The publication of *Dong* et al. described the results of a patient cohort of 29 patients with non-malignant disease undergoing HSCT [median age 6.4 years (range 0.28–21.4)], who were enrolled in two different studies (Marsh et al., 2017; Arnold et al., 2021). Alemtuzumab was given as a total dose of 1 mg/kg divided over days -14 to -10 in study 1 (*n* = 17) and in study 2 as a total dose of 0.5–0.6 mg/kg. For patients in study two who were expected to clear alemtuzumab by day of HSCT to ≤0.15 μg/ml, a top up dose was calculated and given either on day -3 or day -1. The authors concluded that the currently used dosing per kilogram strategy causes uneven exposure of alemtuzumab across different weight and age cohorts. They propose an allometric- or body surface area- based starting dosing regimen in combination with TDM to achieve a recommended therapeutic range of 0.15–0.6 μg/ml on the day of transplantation, which is associated with better HSCT outcomes (less aGVHD and improved lymphocyte recovery) (Dong et al., 2021).

Altogether, based on the above-mentioned publications, it can be concluded that, as for ATG, a more individualized dosing strategy of alemtuzumab could improve HSCT outcomes of patients. Since there are only a few studies published about alemtuzumab PK and PD in pediatric patients, the need for further PK&PD analyses is urgent. Currently, an international multicenter observational trial (ARTIC study) is open for patient inclusion. The aim of this study is to evaluate current clinical practice and develop a population PK model and explore the exposure response for alemtuzumab in children with non-malignant diseases. This model will be used to provide important additional information on alemtuzumab treatments and might support the need for therapeutic drug monitoring.

## Discussion

There is a certain set of criteria to determine which drugs are suitable for TDM. The drug should have a narrow therapeutic index and considerable pharmacokinetic variability between patients. Importantly, there should be a reasonable relationship between plasma concentrations and clinical effects, e.g., efficacy or toxicity. The dose cannot be easily optimized by clinical observation and last, but certainly not less important, a bioanalytical assay should be available. The main focus of this review was to assess whether there is a reasonable relationship between plasma concentrations of the most commonly used conditioning agents prior to pediatric HSCT and clinical effects. Taken all the available evidence into account, Bu fulfills all criteria for TDM at the moment which is also reflected in various study protocols and guidelines (Lankester et al., 2021; Peters et al., 2021). Refinement of exposure targets could further improve results for specific subgroups. For Treo, there seems to be some relationship with clinical outcome, however contrasting results are reported. High exposure increases the risk of skin toxicity and, in one study, an association with mortality is seen (van der Stoep et al., 2017; Chiesa et al., 2020; van der Stoep et al., 2021). A cumulative target concentration of 4,800 mg*h/L is suggested in this particular study. In two other studies, no relationship was seen with EFS and OS. For now, the evidence is not convincingly enough to implement Treo TDM for all pediatric patients undergoing HSCT. For Flu, the same arguments can be made. Although a large retrospective study showed a relationship of Flu exposure and EFS, and suggested an optimal target of 15–25 mg*h/L, other studies did not find a clear relationship (Ivaturi et al., 2017; Mohanan et al., 2017; Chung et al., 2018; Langenhorst et al., 2019). More research on Flu PK/PD may provide further evidence whether Flu TDM is of added value in routine clinical practice. For ATG, almost all studies that studied clinical outcome in relation to exposure (pre- and post HSCT) are done with Thymoglobulin. Delayed immune reconstitution was seen in patients with high (post-HSCT) exposure in several studies, which means that patients could potentially benefit from individualized dosing of ATG (Admiraal et al., 2015a; Admiraal et al., 2016). For ATLG, this still needs to be determined, but a correlation between exposure and outcome seems most likely. High alemtuzumab levels also seem to be correlated with delayed immune reconstitution, but data is still scarce, and more research is needed in order to define a therapeutic target and an optimal dosing regimen (Marsh et al., 2016; Bhoopalan et al., 2020; Arnold et al., 2021; Dong et al., 2021).

While most of the transplant community is convinced that Bu TDM is necessary, it is still not implemented in every transplantation center, since not every center has a bioanalytical assay available, and logistics of shipping samples is challenging or costly. The available population PK models with identified covariates could help centers define individual Bu doses to achieve exposure within the target range without using TDM. However, given the narrow therapeutic index of Bu and the fact that there is still considerable unexplained variability in Bu PK, the use of population PK models in combination with TDM would be the best option. For ATG, the evidence is suggesting that individualized, PK-guided dosing of ATG improves patient outcome, but due to the time-consuming and expensive assays that are currently available, TDM will probably not be an option for most centers at short notice (Keogh et al., 2022). Easy to operate and less expensive bioanalytical assays are warranted to overcome this hurdle. Because the therapeutic index of ATG is likely to be much wider, using population PK models to estimate individual ATG doses would be a feasible option for most transplant centers.

When interpreting the data of all these studies, there are some limitations that must be kept in mind. Some studies only report small patient numbers, which makes it difficult to assess the possible influence of transplantation related covariates on outcome. Also, different combinations of conditioning agents and the addition of serotherapy to the regimen are major factors that influence outcome. Furthermore, due to the constantly evolving field and improvement of the transplant procedures over time, some of the regimens and procedures in the reported studies are already amended or revised/renewed.

As much as we can limit the toxicities of chemo-based conditioning, not all side effects are avoidable. Serious concerns about the long-term effects have driven the search for alternative conditioning regimens. Leukocytolytic monoclonal antibodies can provide a potential alternative to achieve myelosuppression and immunosuppression without the concomitant non-hematological toxicity of chemotherapy. Anti-CD45 monoclonal antibodies target CD45 that are selectively expressed on all leukocytes and hematopoietic progenitors. In a study with high-risk pediatric patients with different inborn errors of immunity (IEI), conditioning with anti-CD45 antibodies in combinations with alemtuzumab, fludarabine and cyclophosphamide resulted in myeloid and lymphoid engraftment (Straathof et al., 2009). Antibodies conjugated with radionuclides, known as radioimmunotherapy, can deliver radiotherapy directly to the surface of the targeted cells. Normal tissue gets spared, making this kind of conditioning a potentially less toxic alternative (Schulz et al., 2011). Promising results with anti-CD117 monoclonal antibody as an alternative for traditional conditioning can possibly change the way we prepare patients for HSCT in the future (Kwon et al., 2019). For an increasing number of pediatric diseases, alternative treatment strategies have become available, such as gene therapy. However, while these therapies are very promising, allogeneic HSCT with the use of “regular” conditioning regimens will still be the first (and sometimes only) option in many diseases. More research regarding the late effects of conditioning is therefore crucial to further optimize combinations and dosing of conditioning regimens.
